# Use of biomarkers for predicting a malignant course in acute ischemic stroke: an observational case–control study

**DOI:** 10.1038/s41598-023-43408-z

**Published:** 2023-09-26

**Authors:** Alexandre Guimarães de Almeida Barros, Lucas Roquim e Silva, Alberlúcio Pessoa, Antonio Eiras Falcão, Luiz Alexandre Viana Magno, Daniela Valadão Freitas Rosa, Marco Aurelio Romano Silva, Debora Marques de Miranda, Rodrigo Nicolato

**Affiliations:** 1 Medical School of Universidade Federal de Minas Gerais, Prof. Alfredo Balena Avenue, 190, Belo Horizonte, 30130-100 Brazil; 2 Medical School of Universidade Estadual de Campinas, R. Vital Brasil, 251, Cidade Universitária, Campinas, 13083-888 Brazil

**Keywords:** Stroke, Predictive markers

## Abstract

Acute ischemic stroke is a sudden neurological event caused by brain ischemia. Patients with large vessel occlusion are at high risk of developing significant cerebral edema, which can lead to rapid neurological decline. The optimal timing for decompressive hemicraniectomy to prevent further brain damage is still uncertain. This study aimed to identify potential predictors of severe brain edema. The data indicate that specific cytokines may help identify patients with a higher risk of developing life-threatening brain swelling in the early phase post-stroke. The association between a positive biomarker and the outcome was calculated, and three biomarkers—S100B protein, MMP-9, and IL-10—were found to be significantly associated with malignant edema. A model was derived for early predicting malignant cerebral edema, including S100B protein and IL-1 beta. These findings suggest that molecular biomarkers related to the ischemic cascade may be a helpful way of predicting the development of malignant cerebral edema in ischemic stroke patients, potentially widening the time window for intervention and assisting in decision-making. In conclusion, this study provides insights into the molecular mechanisms of severe brain edema and highlights the potential use of biomarkers in predicting the course of ischemic stroke.

## Introduction

Acute ischemic stroke is characterized by sudden neurological impairment caused by cerebral ischemia persisting for more than 24 h or by imaging evidence of brain infarction, regardless of symptom duration^1^. Cerebral hypoperfusion due to atherosclerotic disease or embolism is a common underlying cause of stroke. Patients with occlusion of major cerebral vessels, such as the middle cerebral or internal carotid artery, frequently exhibit significant hemispheric infarction, cerebral edema, and rapid neurological deterioration^1–3^. Imaging measures of hypodensity extension and infarct volume are currently the most dependable predictors of a severe course^4–6^. Decompressive hemicraniectomy has been implemented in patients at high risk for significant cerebral edema to prevent further brain damage by reversing mass effect, reducing brain tissue shifts, decreasing intracranial pressure, and improving cerebral perfusion pressure^7–10^.

Several clinical trials have demonstrated the potential advantages of decompressive hemicraniectomy, including reduced mortality rates and improved long-term functional outcomes with a corresponding increase in patients who survive with a minimal-to-moderate disability, particularly in younger individuals^3,7,8,11–16^. Nevertheless, one study has suggested that decompressive hemicraniectomy may produce similar outcomes compared to optimized medical management^17^. Notably, the observed heterogeneity among these trials, such as differences in inclusion criteria, standardized medical approaches, and timing of surgery, may explain the divergent findings. The optimal timing for decompressive hemicraniectomy remains unclear, and further research is required to better understand the extent of cerebral edema following acute ischemic stroke and the criteria for selecting patients for the procedure^10,16^.

Currently, imaging studies represent the primary diagnostic and monitoring tools employed for patients with acute ischemic stroke. Repeated cranial tomography within the first three days after stroke onset can identify the infarction area, the degree of associated brain edema, and the extent of midline shift^4^. However, imaging findings consistent with the development of cerebral edema and mass effect often lag behind the underlying molecular pathways triggered by acute ischemic infarction.

To gain insight into brain swelling associated with ischemic stroke and investigate potential indicators of severe brain edema, we examined molecular markers related to the development of malignant cerebral edema. We hypothesized that molecular biomarkers linked to the established molecular cascade of stroke could predict a malignant course of cerebral edema in patients with an ischemic stroke earlier. Such biomarkers could potentially expand the intervention time window and aid in decision-making.

## Results

### Patient population

**Figure 1:**
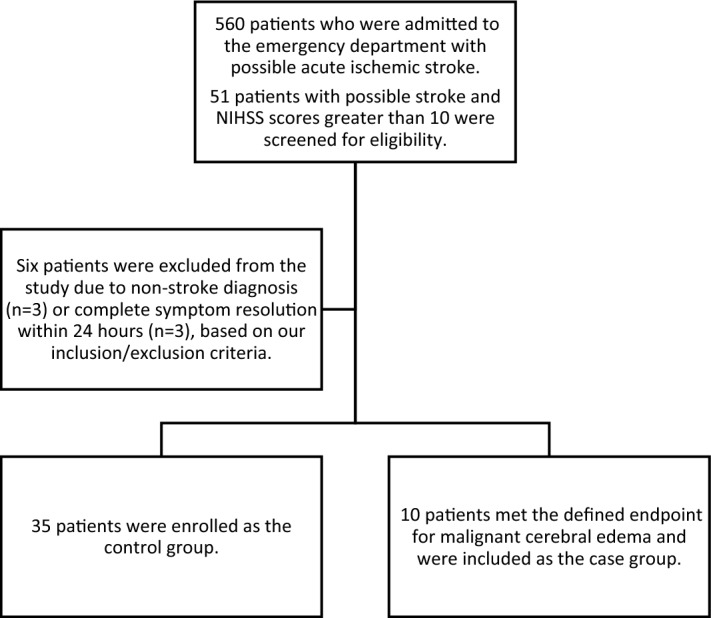
Flowchart of patient recruitment.

A total of 560 patients were admitted to the hospital emergency department with acute ischemic stroke during the study period. We initially assessed 51 patients from this population, but only 45 were eligible for further analysis according to our eligibility criteria (as shown in Fig. 1). The main reason for exclusion was neurological improvement within the first 24 h after enrollment. All patients assessed had their data and outcomes available; no further exclusion was necessary. Blood samples and clinical data were collected at appropriate time points, and patients were followed until they reached the primary outcome or the end of the follow-up period. During the evaluation period, the mortality rate was 7%.

### Demographics and clinical features

The demographics and clinical features of the analyzed population are summarized in Table 1. At admission, the median NIHSS and Glasgow Coma Scale scores were 18 and 14, respectively. Ten patients met the defined endpoint for malignant cerebral edema. They comprised the case group for the study, while the remaining 35 patients were included as the control group for the final analysis. The clinical features, time from first symptoms, and blood draw for cytokine measurements showed no statistical difference between the two groups.

**Table 1:** Baseline characteristics of the study case and control groups.

	Total sample (n = 45)	Malignant stroke group (n = 10)	Control group (n = 35)	*p* value
Age (median (IQR))	70 (60.75–79.25)	71 (54–80)	70 (62.25–79)	0.600
Female (count (%))	23 (51%)	3 (30%)	20 (57%)	0.457
First clinical evaluation data				
DeltaT blood draw (median (IQR), minutes)	600 (493.75–783.75)	725 (475–915)	600 (495.75–770)	0.366
Glasgow coma scale (median (IQR))	14 (–11 to 15)	13.5 (–11 to 15)	14 (11–15)	0.352
Bamford scale (count (%))				
TACS	30 (67%)	8 (80%)	22 (63%)	0.407
PACS	10 (22%)	2 (20%)	8 (23%)	
LACS	5 (11%)	0 (0%)	5 (14%)	
Initial SBP (median (IQR), mmHg)	148.5 (130–170)	150 (143–181)	146.5 (130–170)	0.339
Initial DBP (median (IQR), mmHg)	86.5 (78.5–100)	80 (76–100)	87.5 (80–100)	0.431
Initial MBP (median (IQR), mmHg)	106.15 (96.7–121.65)	101.65 (96.7–128)	106.5 (96.7–120)	0.989
Initial Heart Rate (Median (IQR), bpm)	73 (64.75–85)	72 (66–79)	77 (64.75–85.25)	0.663
Blood Glucose ( Median (IQR), mg/dL)	115 (101.75–144.75)	107 (97.75–146.25)	118 (102.5–145.5)	0.546
Initial NIHSS ( Median (IQR))	18 (13.75–20.25)	19 (17–21)	17 (13–20)	0.626
Venous thrombolysis (rTPa EV) (Count (%))				
Yes	28 (62%)	5 (50%)	23 (66%)	0.366
V enous thrombolysis Delta T (Median (IQR), minutes)	140 (109.75–185.25)	152 (113–187.5)	140 (108–187)	–
Neurovascular risk factors (count (%))				
First 24 h nausea, and vomiting	12 (27%)	4 (40%)	8 (23%)	0.311
Systemic arterial hypertension	33 (73%)	6 (60%)	27 (77%)	0.280
* Diabetes Mellitus*	7 (16%)	2 (20%)	5 (14%)	0.660
Smoking history	15 (33%)	4 (40%)	11 (31%)	0.612
Dyslipidemia	10 (22%)	3 (30%)	7 (20%)	0.502
Chronic renal disease	4 (9%)	1 (10%)	3 (9%)	0.889
Ischemic stroke history	11 (24%)	1 (10%)	10 (29%)	0.228
Hemorrhagic stroke history	1 (2%)	0 (0%)	1 (3%)	0.589
Myocardial infarction history	2 (4%)	0 (0%)	2 (6%)	0.439
Familial history of stroke or myocardial infarction	11 (24%)	3 (30%)	8 (23%)	0.643
Heart failure	15 (33%)	4 (40%)	11 (31%)	0.612
Known atrial fibrillation	11 (24%)	3 (30%)	8 (23%)	0.643
Unknown atrial fibrillation	14 (31%)	3 (30%)	11 (31%)	0.931
Second clinical evaluation data and outcomes				
DeltaT for the second clinical evaluation (median (IQR), minutes)	7073 (5840–7690)	5690 (5237–6233.75)	7200 (6346.25–8472.25)	0.006*
Modified rankin scale (median (IQR))	5 (3.5–5)	5 (5–5.25)	4 (3–5)	0.039*
Second evaluation NIHSS (median (IQR))	14 (4–18)	22 (17.5–28)	10 (3.25–18)	0.096
Cerebral herniation (count (%))	4 (9%)	4 (40%)	0 (0%)	< 0.001*
Anisocoria (count (%))	5 (11%)	5 (50%)	0 (0%)	< 0.001*
Neurologic symptoms worsened during follow-up (count (%))	13 (29%)	9 (90%)	4 (11%)	< 0.001*
Unconsciousness (count (%))	10 (22%)	10 (100%)	0 (0%)	< 0.001*
NIHSS variation between the clinical evaluations (mean (± SD))	− 3.82 (± 8.26)	9.17 (± 8.23)	− 6.06 (± 5.96)	0.030*
Glasgow Outcome Scale (median (IQR))	3.0 (3–4)	2.0 (1–2)	3.0 (3–4)	< 0.001*
Death (count (%))	3 (7%)	3 (30%)	0 (0%)	0.001*
Decompressive craniectomy (count (%))	4 (9%)	4 (40%)	0 (0%)	0.008*
More than 24 h use of EV vasopressor medication (count (%))	1 (2%)	1 (10%)	0 (0%)	0.046*
More than 24 h use of EV hypotensive medication (count (%))	7 (16%)	3 (30%)	4 (11%)	0.109

Noteworthy, despite the absence of any relationship between the eligibility criteria and Bamford classification, some patients in the control group were characterized as having lacunar syndrome (LACS). Data are presented as median and interquartile range (IQR), as count and percentage (%) or as mean ± standard deviation (SD).

Abbreviations: *TACS* total anterior circulation syndrome, *PACS* partial anterior circulation syndrome, *LACS* lacunar stroke syndrome, *SBP* systolic blood pressure, *DBP* diastolic blood pressure, *MBP* mean blood pressure, *mmHg* millimeters of mercury column, *bpm* beats per minute, *NIHSS* national institute of health stroke scale, *rTPa EV* venous recombinant tissue plasminogen activator, *DeltaT* time variation, *EV* endovenous.

*Indicates a *p* value < 0.05.

### Serum cytokine analysis

To investigate the potential for early prediction of severe cerebral edema in ischemic stroke, we measured serum cytokines in the acute phase in enrolled patients, as shown in Table 2. Our findings revealed significantly elevated levels of serum astroglial protein S100B in patients who developed malignant cerebral edema compared to the control group. The median blood concentration of S100B protein was 952.23 pg/mL and 170.47 pg/mL in the case and control groups, respectively.

**Table 2:** Cytokines concentrations at baseline.

Cytokine (pg/mL) (mean (± SD)))	Malignant stroke group	Control group	*p* value
S100B	952.23 (± 1158.33)	170.47 (± 114.87)	0.007*
TNF-alpha	11.44 (± 6.54)	8.39 (± 3.23)	0.262
IL-6	34.66 (± 63.9)	12.25 (± 12.88)	0.298
MMP-9	60,920.5 (± 43,730)	33,617.54 (± 30,179)	0.065
IL-10	30,460.3 (± 21,864.85)	16,808.8 (± 15,089.48)	0.065
IL-1beta	10.55 (± 5.57)	7.32 (± 2.63)	0.083

Data are presented as mean ± standard deviation (SD) in pg/mL.

*Indicates a *p* value < 0.05.

### AUROC analysis for cytokines

Using the composite of primary endpoints as the dependent target condition, we calculated C-index statistics for each measured cytokine, as presented in Table 3. The results indicated that S100B protein had the highest AUROC of 0.776 (95% CI 0.578–0.973), followed by MMP-9, IL-10, and IL-1 beta. Cut-off points were established for each biomarker, and Table 4 shows the accuracy and likelihood ratios for the outcome. We found that IL-6 and S100B exhibited an excellent negative LR using the set cut-off points, while S100B showed a fair positive LR. These results suggest that both cytokines may predict malignant edema development.

**Table 3:** C-index statistics for each measured cytokine using set cut-off points.

Cytokine	Cut-off (pg/mL)	AUROC (95% CI)	*p* value
S100B	329.42	0.775 (0.626–0.886)	0.008*
TNF-alpha	6.75	0.618 (0.461–0.759)	0.32
IL-6	5.15	0.611 (0.454–0.752)	0.29
MMP-9	22,407	0.694 (0.539–0.822)	0.072
IL-10	11,204	0.694 (0.539–0.822)	0.072
IL-1beta	6.74	0.682 (0.527–0.813)	0.089

*Indicates a *p* value < 0.05.

**Table 4:** Accuracy and likelihood ratios for the outcome based on defined cut-offs for each measured cytokine.

Biomarkers	Cut-off point (pg/mL)	Sensitivity (%)	Specificity (%)	LR+	LR−
S100B protein	> 329.42	70	85.7	4.9	0.35
TNF-alfa	> 6.75	80	48.6	1.56	0.41
IL-6	> 5.15	100	28.6	1.40	0.00
MMP-9	> 22,407	80	57.1	1.87	0.35
IL-10	> 11,204	80	57.1	1.87	0.35
IL-1beta	> 6.74	70	62.9	1.88	0.48

*LR* likelihood ratio.

### Odds ratios for biomarkers

Odds ratios were calculated to determine the association between a positive biomarker and the composite outcome. The cut-off values for positivity were derived from AUROCs, achieving the best balance between sensitivity and specificity (Tables 3 and 4). Three biomarkers, S100B protein, MMP-9, and IL-10, showed significant odds of malignant edema occurrence based on the established cut-off values with ORs of 14 (95% CI 2.11–104.84; *p* = 0.0004), 5.33 (95% CI 0.85–56.62; *p* = 0.038), and 5.33 (95% CI 0.85–56.62; *p* = 0.038), respectively (Table 5). The statistically significant *p* values of 0.038 for MMP-9 and IL-10 justify their association with malignant edema, despite the 95% confidence intervals crossing 1. We also analyzed the association of various clinical features with the primary endpoint, including initial NIHSS (National Institute of Health Stroke Scale) score, history of hypertension, diabetes mellitus, smoking status, dyslipidemia, chronic kidney disease, history of previous ischemic or hemorrhagic stroke, acute myocardial infarction, history of stroke or acute myocardial infarction in the family, past or presence of atrial fibrillation in the initial electrocardiogram, among others. No other clinical feature analyzed had a meaningful association with the primary endpoint.

**Table 5:** Association between biomarkers and outcome.

Biomarker	Odds ratio	Confidence interval 95%	*p* value
S100B protein	14.00	2.11–104.84	0.0004*
TNF alfa	3.77	0.60–40.37	0.106
MMP-9	5.33	0.85–56.62	0.038*
IL-10	5.33	0.85–56.62	0.038*
IL-1Beta	3.94	0.71–26.98	0.065

The best cut-off point for each biomarker was used to categorize patients as positive or negative for the outcome, and the odds ratio for the outcome was determined using logistic regression analysis.

*Indicates a *p* value < 0.05.

### Logistic regression model for prediction

Our research indicates that biomarkers hold promise in predicting the likelihood of life-threatening brain swelling during the early phase following a stroke. To develop an early prediction model for malignant cerebral edema, we conducted a logistic regression analysis to determine the capability of biomarkers to predict the onset of malignant cerebral edema in the early stages after a stroke. The model predictions generated a contingency table (Table 6), which allowed us to evaluate the model's performance. The model exhibited promising performance, with a positive predictive value (PPV) of 97.1%, negative predictive value (NPV) of 60%, sensitivity of 89%, and specificity of 85%. However, further validation using a larger and more diverse cohort is warranted to ensure the reliability and generalizability of our findings. Overall, the combination of S100B protein and IL-1 beta holds potential as valuable biomarkers for early prediction of malignant stroke edema, which could have significant implications for clinical decision-making and patient outcomes.

**Table 6:** Performance of the logistic regression model for early prediction of malignant cerebral edema.

		Malignant stroke edema		
		Positive	Negative	
Model prediction of malignant stroke edema	Positive	34	1	PPV 97.1%
	Negative	4	6	NPV 60%
		Sensitivity 89%	Specificity 85%	

*PPV* positive predictive value, *NPV* negative predictive value.

## Discussion

Our study has demonstrated the potential of three serum biomarkers, obtained within 20 h of stroke symptom onset, to serve as prognostic indicators for the emergence of life-threatening cerebral edema in patients with large cerebral artery occlusion with fair diagnostic accuracy. The primary outcome exhibited a significant association with S100B protein, while IL-10 and MMP-9 displayed a noteworthy trend, albeit not reaching statistical significance. Additionally, S100B protein and IL-6 exhibited a fair negative likelihood ratio within the studied population, offering promise for streamlining diagnostic and therapeutic approaches in routine clinical practice. Notably, during the early phase of stroke, none of the baseline clinical features, including disability scales (modified Rankin scale) or symptom scales (National Institute of Health Stroke Scale), exhibited similar predictive capabilities as the analyzed cytokines.

Our research also underscores the promising role of biomarkers in the early prediction model of severe cerebral edema. We have constructed a predictive model for malignant cerebral edema using logistic regression. This model, which integrates S100B protein and IL-1 beta, demonstrated a fair accuracy, correctly identifying 89% of patients in our sample who reached the primary endpoint. In this model, a positive prediction indicates a heightened likelihood of patients developing malignant stroke edema derived from their specific biomarker levels. Conversely, a negative prediction signals a diminished risk. It is vital to note that elevated levels of both biomarkers do not exclusively determine a positive prediction but rather their synergistic impact, which may also account for other pertinent variables as determined by the logistic regression analysis. Notably, the S100B protein exhibited a significant statistical impact in our study compared to IL-1 beta when considered individually. However, its contribution in tandem with the model was significant, enhancing its efficacy in risk assessment. The model delivered a positive predictive value of 97.1% and a negative predictive value of 60%, alongside 89% sensitivity and 85% specificity. These findings emphasize the potential benefits of integrating the S100B protein and IL-1 beta into risk stratification models, fostering more precisely early intervention strategies for individuals at high risk of experiencing cerebral edema post-stroke. To solidify these initial findings and to ascertain their clinical relevance definitively, extended research with more extensive and varied cohorts is imperative.

Inflammation plays a critical role in the pathogenesis of ischemic stroke, and numerous studies have highlighted the rapid increase in inflammatory cytokines following stroke onset. The selection of specific inflammatory markers in our study was driven by the growing body of evidence suggesting their involvement in the pathophysiology of ischemic stroke and their potential role in predicting malignant brain edema^2,18–25^. We chose to investigate interleukin-1 beta (IL-1β), interleukin-6 (IL-6), tumor necrosis factor-alpha (TNF-α), and matrix metalloproteinase-9 (MMP-9) due to their well-established roles as pro-inflammatory mediators. These cytokines are known to be rapidly upregulated in the brain after a stroke and can contribute to blood–brain barrier disruption, leukocyte infiltration, and neuronal damage^18,19,21,25^. Additionally, we explored the potential of the anti-inflammatory marker interleukin-10 (IL-10) to provide a balanced perspective on the immune response following stroke. IL-10 is considered an anti-inflammatory cytokine that can downregulate the production of pro-inflammatory molecules, and its levels may reflect the brain's attempt to modulate the inflammatory response and limit secondary damage^18,19,21,25^. In addition, S100B protein, a calcium-binding protein predominantly expressed in astrocytes, has emerged as a potential biomarker for cerebral injury and brain edema^20,22–24^. S100B can be released into the bloodstream following ischemic stroke due to glial cell activation and blood–brain barrier disruption. Studies have shown that increased serum levels of S100B are associated with infarct volume, risk of hemorrhagic transformation, and functional outcomes in stroke patients, highlighting its potential as a marker for predicting the development of malignant brain edema^20,22–24^. Our findings are consistent with these observations, as we identified elevated levels of three cytokines within 20 h of stroke symptom onset in patients who later developed malignant cerebral edema. Our data analysis using C-statistics revealed that S100B protein had the best predictive performance, yielding a sensitivity of 70% and specificity of 85.7%, with a positive likelihood ratio of 4.9 and a negative likelihood ratio of 0.35 in our cohort. The clinical significance of our findings is exciting in guiding the decision-making process for decompressive surgery in individuals with large ischemic areas and high symptom burden but inconclusive imaging studies.

The existing literature lacks a well-established approach for assessing biomarkers as predictors of malignant cerebral edema development. One prior study found an association between early S100B protein serum levels and life-threatening cerebral edema, which supports our findings^24^. They observed that serum S-100B levels, assessed within 24 h of symptom onset, possess an independent association with symptomatic intracranial hemorrhage and symptomatic brain edema in patients with acute ischemic stroke. Another study showed a correlation between S100B levels and clinical outcomes at 90 days post mechanical thrombectomy underscoring the potential of S100B as a reliable predictor of long-term patient prognosis^22^. In contrast, another group examined the correlation of eight biomarkers, including IL-6, IL-10, MMP-9, and TNFα, and discovered a strong relationship between MMP-9 levels and malignant cerebral edema development, with a sensitivity of 90% and specificity of 100%, which was not replicated in our cohort^27^. The difference may be explained by younger patients and more TACS syndrome in their sample, resulting in a different cytokine pattern.

Our study has several limitations that need to be taken into account. Firstly, the small sample size may have increased the level of uncertainty and reduced the power to detect significant associations for some of the biomarkers. Additionally, the pragmatic design of our study, which aimed to reflect real-world scenarios by including patients with diverse neurovascular risk factors and ages, may have introduced bias. An aspect worth highlighting is the inclusion of lacunar syndromes in our cohort. In the realm of ischemic strokes, lacunar syndromes are traditionally categorized as resulting from small subcortical infarcts, with a milder clinical presentation compared to larger territorial strokes. However, our objective was to ensure that no subset of patients with severe clinical presentations was overlooked, irrespective of the conventional stroke subtype classifications. This decision may raise questions regarding the spectrum of stroke presentations, and while it serves to enhance the comprehensiveness of our findings, it is vital to interpret the results with this consideration in mind. Another noteworthy point is the statistically significant *p* values observed for MMP-9 and IL-10 measurements. While these *p* values of 0.038 for MMP-9 and IL-10 support their association with malignant edema, the 95% confidence intervals for these associations cross 1. The overlapping 95% CI suggests that while these associations are statistically significant, the actual effect size of these biomarkers may vary within the provided range. This variability emphasizes the need for caution when interpreting the magnitude of these associations. It is crucial to base interpretations on statistical significance and consider the clinical relevance and biological implications of these findings. Although we took steps to minimize potential bias through standardization, normalization, and following a rigid protocol, the possibility of bias cannot be ruled out entirely. Despite these limitations, our findings provide important insights into the potential role of biomarkers in predicting the course of acute ischemic stroke. They should be considered foundational hypotheses for future studies that utilize more significant and more representative samples.

In conclusion, there has been a growing interest in exploring the roles of biomarkers in ischemic stroke and their potential to predict patient outcomes. Such biomarkers are promising predictors of disease burden and clinical outcomes, providing clinicians with valuable risk stratification and treatment decision-making tools. Identifying elevated cytokine levels within the early hours of stroke onset offers valuable insights into disease progression and may aid in tailoring treatment strategies. The S100B protein, as a potential biomarker in our present study, aims to shed light on its role in predicting edema formation and its significance in clinical decision-making. These findings underscore the growing importance of biomarkers in ischemic stroke research, which may eventually lead to personalized therapeutic strategies and improved patient outcomes. However, further research and validation through large-scale prospective studies are warranted to fully establish these biomarkers' clinical utility and generalizability in ischemic stroke management.

## Methods

### Study design and setting

We conducted an original observational prospective cohort study utilizing a case–control design within a single reference hospital for acute stroke care in Belo Horizonte, Brazil. This framework allowed us to compare biomarkers in acute stroke patients who developed endpoints related to intracranial hypertension and control participants. The Odilon Behrens Municipal Hospital Ethics Committee approved the research protocol (approval number CAAE: 58195316.0.0000.5129), and all participants or their legally authorized surrogates provided written informed consent to participate in the study. All methods described in this study were conducted in strict accordance with the relevant guidelines, regulations, and the Declaration of Helsinki, ensuring the ethical principles and welfare of the participants were upheld throughout the research process. The recruitment period covered October 2016 to June 2017; patients were followed up for twenty days from admission.

### Recruitment and eligibility criteria

The study enrolled sequential patients admitted to the hospital stroke unit which met the following inclusion criteria: a confirmed stroke diagnosis by both the attending neurologist and research team member, with symptoms onset within 18 h before admission. Those patients were screened and enrolled first and excluded if their symptoms rapidly resolved within 24 h after entry. Additionally, patients needed to have an NIHSS score of 10 or higher and provide signed informed consent. Patients were also excluded if they had experienced coma or seizure activity during the stroke symptoms, had a fever seven days before the stroke or three days following admission, or had immunosuppression. Patients were evaluated for eligibility only if they had complete electronic records. Noteworthy, the eligibility criteria did not include thrombolysis or thrombectomy.

### Data collection and follow-up

Following enrollment, blood samples and clinical data were collected. Blood samples were collected within 20 h after the onset of stroke symptoms in eligible patients. Clinical status and outcomes were assessed in a second clinical visit three to seven days later and reassessed remotely after twenty days. This timeframe was chosen based on the kinetics of biomarkers and the clinical relevance of the results.

After recruitment and follow-up, patients were divided into two groups based on whether they met specific endpoints. The primary outcome measure was the occurrence of clinically relevant intracranial hypertension. The surrogate markers employed for its characterization included the development of malignant stroke, the implementation of decompressive hemicraniectomy, and a reduction of four points in the Glasgow Coma Scale without any discernible cause between the initial and subsequent clinical evaluations. These indicators were confirmed by both the neurologist and research team member within 20 days of admission. Malignant stroke was defined as the presence of any of the following: signs of middle cerebral artery (MCA) infarct with an NIHSS score of 15 or higher and loss of one point in field 1a at any point during follow-up, detection of perfusion compromise and edema with mass effect covering at least two-thirds of middle cerebral artery territory on an image with ventricular compression or midline shift of the septum pellucidum greater than 5 mm, or neurological deterioration without an apparent cause within five days of hospital admission.

### Measurement of biomarkers

The expression levels of six cytokines, namely S100B protein, MMP-9, TNF-alpha, IL-1β, IL-10, and IL-6, were quantified using the Immunobead assay Human Magnetic Luminex Screening Assay (R&D Systems). Plasma samples were separated from blood and stored at − 80 °C until analysis. Duplicate measurements were taken for each sample, with approximately 50 beads collected per assay for each well. The median fluorescence intensity for each marker was calculated and converted to concentrations (pg/ml) using the standard range equation derived from Milliplex Analyst Software. Samples not thawed or refrozen before were analyzed in a single assay to minimize interassay variation.

### Analysis and statistical methods

We assessed the ability of the measured cytokines to predict the development of the defined target condition during the study period by examining their discrimination performance. Discrimination refers to the capability of an independent variable to differentiate between those who do and do not develop the outcome of interest. We used the concordance index (c-index) statistic to estimate discrimination and calculated the area under the receiver operating characteristic curve (AUROC) with intracranial hypertension surrogates as the binary endpoints. A value of 0.5 for AUROC indicates that the predictor cannot distinguish between positive and negative outcomes, representing chance. On the other hand, a value of 1 represents perfect discrimination. Discrimination was categorized according to AUROC values: 0.90–1 excellent, 0.80–0.90 good, 0.70–0.80 fair, 0.60–0.70 poor, and 0.50–0.60 fail. We used the DeLong method to determine whether there were significant differences between distinct models.

To minimize potential sources of bias, we followed strict inclusion and exclusion criteria during patient enrollment. We also ensured that the attending neurologist and research team member confirmed the stroke diagnosis and endpoint criteria. Additionally, we collected and analyzed biological samples and clinical data in a standardized manner. Finally, we followed up with patients for 20 days to confirm endpoint criteria and re-evaluate clinical status, reducing the potential for misclassification bias. Overall, these efforts helped to increase the validity and reliability of our results and minimize potential sources of bias.

The statistical analyses were conducted using two software programs, STATA (version 14.0) and SPSS (version 22.0.0.0). Univariate analysis was performed for continuous and categorical variables to evaluate their association with the endpoint of interest. Continuous variables were reported as mean and standard deviation or median and interquartile ranges based on their distribution. Categorical variables were presented as count and proportion. Odds ratios were calculated after adjusting for illness severity, using a case–control matching strategy with NIHSS scores as specific criteria. A p-value of less than 0.05 (two-tailed) was considered statistically significant.

The execution of this study adhered to the established medical standards and followed the STROBE guideline for observational studies^28^.

## Data Availability

The data of this study can be obtained from the corresponding author upon reasonable request and compliance with privacy and ethical standards. All pivotal data are included within the manuscript.
